# Erythropoiesis and Iron Sulfur Cluster Biogenesis

**DOI:** 10.1155/2010/329394

**Published:** 2010-08-31

**Authors:** Hong Ye, Tracey A. Rouault

**Affiliations:** Molecular Medicine Program, Eunice Kennedy Shriver National Institutes of Child Health and Human Development at (NIH), 9000 Rockville Pike, Bethesda, MD 20892, USA

## Abstract

Erythropoiesis in animals is a synchronized process of erythroid cell differentiation that depends on successful acquisition of iron. Heme synthesis depends on iron through its dependence on iron sulfur (Fe-S) cluster biogenesis. Here, we review the relationship between Fe-S biogenesis and heme synthesis in erythropoiesis, with emphasis on the proteins, GLRX5, ABCB7, ISCA, and C1orf69. These Fe-S biosynthesis proteins are highly expressed in erythroid tissues, and deficiency of each of these proteins has been shown to cause anemia in zebrafish model. GLRX5 is involved in the production and ABCB7 in the export of an unknown factor that may function as a gauge of mitochondrial iron status, which may indirectly modulate activity of iron regulatory proteins (IRPs). ALAS2, the enzyme catalyzing the first step in heme synthesis, is translationally controlled by IRPs. GLRX5 may also provide Fe-S cofactor for ferrochelatase, the last enzyme in heme synthesis. ISCA and C1orf69 are thought to assemble Fe-S clusters for mitochondrial aconitase and for lipoate synthase, the enzyme producing lipoate for pyruvate dehydrogenase complex (PDC). PDC and aconitase are involved in the production of succinyl-CoA, a substrate for heme biosynthesis. Thus, many steps of heme synthesis depend on Fe-S cluster assembly.

## 1. Erythropoiesis

Erythropoiesis, the manufacture of red blood cells (or erythrocytes), mainly occurs within bone marrow in human adults, for review see [1]. In erythropoiesis, there is a stepwise differentiation of cell types, beginning with multipotent hematopoietic stem cells which successively mature into common myeloid progenitor cells, proerythroblasts, erythroblasts, and finally into mature erythrocytes [2]. Erythropoiesis is stimulated by the hormone, erythropoietin (EPO), for review see [3], which enhances proliferation and differentiation of the erythroid cells by blocking apoptosis of erythroid progenitors, as is reviewed elsewhere, for review see [4–8].Hemoglobinization results from the production of hemoglobin, which requires synthesis of heme. Heme is synthesized by an eight step enzyme-catalyzed pathway,in which the final step is the insertion of an iron into protoporphyrin IX to form a protoheme, for review see [9, 10]. The substantial manufacture of heme for hemoglobin in red blood cells consumes 70% of body iron in humans. Iron homeostasis during erythropoiesis is highly regulated to synchronize synthesis of heme and globin and to avoid the potential toxicity caused by accumulation of excess iron or heme.

## 2. Systemic Iron Metabolism and Regulation of Hepcidin Expression by EPO and Other Factors

Iron in food is absorbed in the duodenum, from which it is released into the circulation via ferroportin, the iron exporter on basolateral membranes of duodenal enterocytes. Most of the daily iron supply in the human body comes from phagocytosis of senescent red blood cells by macrophages in the spleen, liver, and bone marrow. Macrophages recycle iron by metabolizing heme and releasing the free iron into the circulation via the membrane-bound ferrous iron transporter, ferroportin [11–13]. The ferroportin-mediated release of iron is therefore a key regulation point of systemic iron metabolism. Hepcidin is a small peptide synthesized mainly in the liver that modulates the abundance of ferroportin at the cellular membrane of cells that release iron, for review see [14–16]. Hepcidin is the master regulator of systemic iron homeostasis: low levels of hepcidin increase iron release into plasma, whereas high hepcidin levels decrease iron release into plasma. The transcription of hepcidin is complex and is finely tuned by a number of different signal transduction pathways, for review see [14, 17–19]. To coordinate iron metabolism to meet the demands of erythropoiesis, hepcidin expression is regulated by EPO, the erythropoiesis stimulator, and also possibly by growth differentiation factor 15 (GDF15) and twisted gastrulation (TWSG1), soluble peptides which are directly produced by erythroblasts [20, 21]. In cultured liver cells (primary hepatocytes and HepG2), hepcidin transcription is regulated by EPO, which mediates its effect through EPO receptor signaling and C/EBP transcription factor [22]. GDF15 secretion from maturing erythroblasts may inhibit hepcidin mRNA expression in hepatocytes, which would therefore allow more release of iron into plasma from the duodenum and macrophages to support erythropoiesis. However, this potential role of GDF15 remains unproven, as GDF15 has failed to suppress hepcidin expression in cellular models [23, 24]. In thalassemia syndromes, GDF15 is overexpressed, and its proposed repression of hepcidin expression leads to iron overload [20]. TWSG1 protein, which is also expressed by erythroblast cells, may regulate hepcidin expression together with GDF15 by interfering with BMP-mediated hepcidin expression [21], or it may act independently of GDF15.

## 3. Effects of Iron Homeostasis on Erythropoiesis

Cellular iron homeostasis in mammals is primarily regulated by the IRP/IRE system, which operates mainly at the posttranscriptional level. Mammalian cells express two iron regulatory proteins (IRPs), including IRP1 (annotated as Aco1 in genome) and IRP2 (annotated as Ireb2 in genome, but commonly referred to as IRP2), for review see [25, 26]. IRP1 protein functions as a cytosolic aconitase when it ligates a [4Fe-4S] cluster whereas it is activated to bind to RNA stem-loops known as iron-responsive elements (IREs) when it lacks an Fe-S cluster. IRP2 is degraded in iron replete conditions whereas it is stabilized in cells that are iron-depleted, and stabilized IRP2 acts as a second IRE-binding protein. The iron-responsive element (IRE) consists of a conserved loop (5′-CAGUGN-3′) at the end of a base-paired helical stem that is interrupted by an unpaired “bulge” cytosine. IREs are usually found in the untranslated regions (UTR) of various mRNAs. When IRP proteins bind to an IRE in 5′UTR they inhibit translation whereas then they bind IREs in 3′UTR of the TfR1 transcript, they stabilize the mRNA. 

 Erythropoiesis depends on ample iron supplies, and the process of erythropoiesis is regulated in several ways by iron metabolism. Erythropoiesis is driven by EPO, a hormone synthesized mainly in renal interstitial cells. Hypoxia inducible factor 2*α* (HIF2*α*) has a major role in the transcriptional activation of EPO, as it binds to the hypoxia-responsive element (HRE) of the EPO gene, and activates EPO transcription [19, 27–30]. Interestingly, HIF2*α* contains a 5′IRE in its transcript, and therefore HIF2*α* protein translation can be repressed when IRE-binding activity is increased, and this effect seems to be mainly mediated by IRP1 [31, 32]. In hypoxic cells, both HIF1*α* and HIF2*α* proteins are stabilized, and transcription of their target genes increases [32]. However, in cells that are also iron deficient, translation and synthesis of HIF2*α* would be expected to be repressed by IRP binding. Although IRP-dependent repression of HIF2*α* has not been formally demonstrated to occur in animal models, the potential for repression of HIF2*α* translation by IRP1 has been demonstrated *in vitro* [32]. Thus, it is likely that the Fe-S protein, IRP1, has an important role in regulating expression of EPO.

 More directly, erythroblasts are themselves significantly dependent on proper iron homeostasis controlled by IRP2 [33, 34] and on successful iron acquisition mediated by transferrin receptor 1 (TfR1) [35] and mitoferrin-1 (or SLC25A37) [36, 37], as shown by anemias that develop when they are deficient in model organisms. IRP2 is the second iron regulatory protein in mammals, which regulates cellular iron homeostasis by binding to transcripts that contain IREs, similar to IRP1 [25] and the IRP2−/− deletion mouse model manifests microcytic anemia. Studies to address the cause of anemia have revealed that TfR1 expression in erythroid precursors of IRP2−/− mice is reduced, and bone marrow iron stores are absent [33], which could interrupt erythropoiesis by limiting iron availability. 

 The important role of TfR1 in iron acquisition by erythroblasts is also supported by studies in the zebrafish model system. Zebrafish expresses two TfR1 genes, TfR1a and TfR1b [35]. TfR1b is expressed primarily in nonerythroid tissues, and genetic ablation of TfR1b causes growth retardation and brain necrosis without adversely affecting hemoglobinization. In contrast, TfR1a is expressed specifically in erythroid precursor cells, and its ablation causes hypochromic microcytic anemia [35]. Since mammals express a single TfR1 gene ubiquitously, which is responsible for transferrin iron uptake in all tissues including erythroid tissues, it is not surprising that disruption of the TfR1 gene in mice affects both erythropoiesis and neurologic development, and deletion of TfR1 in mice is embryonically lethal [38]. 

 Mitoferrin 1 is the principle iron importer on the inner membrane of mitochondria for erythroblasts. It is highly expressed in hematopoietic tissues, and deficiency of mitoferrin 1 impairs iron incorporation into heme, resulting in hypochromic anemia and erythroid maturational arrest in zebrafish [36, 37]. The role of mitoferrin (Mfrn) has been studied in erythroblasts generated from Mfrn−/− murine embryonic stem cells, which showed maturation arrest with severely impaired incorporation of ^55^Fe into heme [36].

## 4. Iron Sulfur Cluster (Fe-S) Biogenesis for Erythropoiesis

Iron sulfur clusters (Fe-S) are synthesized in human cells by a mitochondrial machinery and also by an independent cytosolic machinery, which involve at least 20 proteins in total, for review see [39]. In mitochondria, ISCS and ISD11 form a complex of cysteine desulfurase to provide the sulfur needed for initial Fe-S formation. It is thought that frataxin provides the iron by binding iron loosely to an acidic ridge [40]. Fe-S clusters are assembled upon scaffold proteins, which include ISCU [41], NFU [42], and ISCA [43]. In cytosol, the cytosolic forms of ISCS and ISD11 (c-ISCS and c-ISD11) provide sulfur [44], and iron may be acquired from the cytosolic iron pool, perhaps aided by a chaperone or cytosolic frataxin [45, 46]. In the cytosol, clusters are assembled upon various scaffolds including c-ISCU, c-NFU, c-ISCA, IOP1, for review see [39, 47], and NBP35 [48]. Under conditions that impair mitochondrial Fe-S cluster synthesis, iron is imported into mitochondria with high priority, which in turn results in cytosolic iron deficiency and impairment in cytosolic Fe-S cluster synthesis [41, 44, 49].

 As described above, IRP1 is a dual functional enzyme, which is activated when deprived of its [4Fe-4S] cluster to bind to IRE elements in mRNAs and regulate protein translation. Due to the importance of IRP proteins in iron homeostasis and the involvement of Fe-S clusters in modulating IRP1 activity, the process of Fe-S cluster biogenesis is actually central to the regulation of mammalian cellular iron homeostasis [39]. Defects in human Fe-S cluster biogenesis cause many different diseases, including anemia [25, 47, 50]. Recent studies have revealed that a number of proteins in mitochondrial Fe-S cluster synthesis are required for successful erythropoiesis, including ABCB7, GLRX5, ISCA1-2, and C1orf69, the mammalian ortholog of Iba57.

 ABCB7 is an ATP-binding cassette (ABC) transporter located on the inner membrane of mitochondria, for review see [51–53], which is essential to heme synthesis and erythropoiesis, as revealed by development of sideroblastic anemia in patients with ABCB7 mutations [54–56]. The ABCB7 deficiency results in iron accumulation in mitochondria, reduced heme synthesis in erythrocytes and ineffective erythropoiesis. Perhaps due to its high expression in cerebellum in addition to bone marrow, patients with ABCB7 deficiency also have ataxia [57, 58]. Atm1, the ABCB7 homologue in yeast, has been thought to be a member of the proposed Fe-S cluster export machinery in mitochondrial membranes. The compound exported by Atm1 was originally hypothesized to be an Fe-S cluster, for review see [52, 59]. As research progressed, and Fe-S synthesis proteins were identified in the cytosol, it was hypothesized that the iron for cytosolic Fe-S assembly was acquired from the cytosol, but that the Atm1 substrate contained a specific type of sulfur that was required for cytosolic Fe-S assembly, for review see [60]. In human cells, although its activity is not required for Fe-S cluster biogenesis in mitochondria, the unknown substrate transported by ABCB7 appears to be required for the maintenance of iron homeostasis in cytosol, which may in turn affect the Fe-S cluster biogenesis process in cytosol [39]. Another possibility is that the product exported by ABCB7 perhaps represents a gauge of mitochondrial iron status that contributes to regulation of mitochondrial iron homeostasis. When production or export of this unknown ABCB7 substrate is disturbed, the cell responds as though mitochondria were iron depleted, and efforts to rectify the misperceived state of mitochondrial iron depletion result in actual mitochondrial iron overload and cytosolic iron deficiency [58, 61, 62].

 ABCB7 function is somehow required for erythropoiesis, as ABCB7 deficient animals develop anemia. But it is thus far unclear at which step ABCB7 function affects heme synthesis. It has been suggested that ABCB7 may physically interact with ferrochelatase and somehow support its activity [54]. Another possible point where ABCB7 may exert its effect is upon erythroid ALAS2, the enzyme that catalyzes the first step of heme biosynthesis, which contains a 5′ IRE in its transcript. ABCB7 deficiency activates the IRE binding activity of IRP proteins in cytosol, which in turn may inhibit the translation of ALAS2 [62]. The molecule exported by ABCB7 does not necessarily have to be an Fe-S cluster or its components. Based on the finding that Mdl1, an ABC7-like transporter, is actually a mitochondrial peptide exporter [64], and that the substrate of Atm1 proteins is cysteine rich [65], it is possible that this substrate may be a cysteine-rich small peptide, which signals the rest of the cell about the mitochondrial iron status. It would also be possible that the Atm1 substrate is a sulfur compound in yeast, which may combine with iron to form Fe-S clusters in cytosol [60]. 

**Figure 1:**
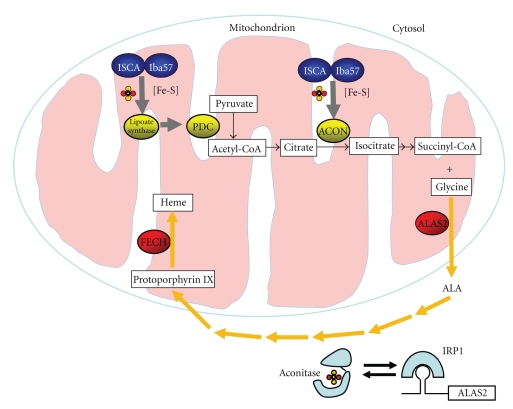
The Fe-S biogenesis proteins, ISCA and C1orf69 (Iba57 homologue), may impact heme synthesis by affecting the production of succinyl-CoA, a substrate of heme synthesis. This proposed role is highly hypothetical and has not been shown in organisms that perform erythropoiesis. The ISCA- C1orf69 complex provides Fe-S clusters for lipoate synthase as suggested in yeast [63], which produces lipoate for a subunit of the pyruvate dehydrogenase complex (PDC). PDC converts pyruvate into acetyl-CoA, which enters the citric acid cycle to form citrate. The ISCA- C1orf69 complex provides the Fe-S cluster for mitochondrial aconitase, which converts citrate to isocitrate, which leads to synthesis of succinyl-CoA, a substrate for the first step in heme biosynthesis. All proteins are in the matrix. Other Fe-S proteins important in heme biosynthesis are ferrochelatase, which is unstable without its [2Fe-2S] cluster, and IRP1, which represses synthesis of ALAS2 when it lacks an Fe-S cluster. In the heme synthesis pathway, succinyl-CoA and glycine are condensed into 5-aminolevulinic acid (ALA). The subsequent six steps take place either in cytosol or in the intermembrane space of mitochondria. The last step is the insertion of a ferrous iron into protoporphyrin IX by ferrochelatase (FECH) to result in heme formation.

 Heme biosynthesis is achieved by eight enzyme-catalyzed steps (Figure 1). The first step of heme synthesis, the condensation of succinyl-CoA and glycine into 5-aminolevulinic acid (ALA), is catalyzed by ALAS2 in the mitochondrial matrix of erythroid precursor cells. ALA is exported to the cytosol, and the subsequent six steps of heme synthesis take place either in the cytosol or in the intermembrane space of mitochondria. The heme intermediate, protoporphyrin IX, is imported into mitochondria. In the last step, ferrochelatase inserts an iron into protoporphyrin IX to produce heme [10, 66].

 Frataxin is thought to be the potential iron donor for Fe-S cluster biogenesis in mitochondria [51], and loss of functional frataxin alters heme synthesis pathway in mammalian cells [67] and in a mouse model for the human disease, Friedreich ataxia [68]. Frataxin may also provide iron to the ferrochelatase-catalyzed last step in heme biosynthesis, in which ferrous iron is inserted into protoporphyrin IX [69, 70]. However, Friedreich ataxia patients do not demonstrate significant anemia, suggesting frataxin is not essential for heme synthesis and erythropoiesis, or that frataxin-deficiency is not present in erythropoietic tissues of Friedreich ataxia patients. In a frataxin deficient mouse model and in Friedreich ataxia patient lymphoblast cells, heme production was not reduced, but the heme derivatives, mitochondrial heme A and heme C levels were decreased [67]. This may be because frataxin is required to assemble Fe-S clusters for ferredoxin. In *S. cerevisiae,* the vinyl group at C-2 of heme B is farnesylated to generate heme O. Subsequently, the methyl group at C-8 of heme O is hydroxylated by monooxygenase that depends on ferredoxin to generate heme A. [71]. The decrease in [2Fe-2S]-ferredoxin activity caused by frataxin deficiency may interrupt the conversion of heme O to heme A. 

 GLRX5, the human ortholog of yeast GRX5, for review see [72–74], represents a member of a family of highly conserved monothiol glutaredoxins that are essential to Fe-S cluster biogenesis in yeast, plants, and mammals [75–77], and GLRX5 deficiency caused sideroblastic anemia in an Italian patient [74]. Thus far, several monothiol glutaredoxin proteins have been shown to assemble an intermolecular [2Fe-2S] cluster that is ligated between a glutaredoxin dimer and by two glutathione peptides *in vitro* [78–80], and it has been suggested that GRX5 homologues, including the human GLRX5, could serve as an alternative scaffold protein that may deliver Fe-S clusters to a specific subgroup of target proteins [81]. As previously discussed, the molecule exported by ABCB7 is likely produced or dependent on the mitochondrial Fe-S biogenesis machinery. We hypothesize that this unknown molecule may signal the rest of the cell about the status of iron in mitochondria and lead to transcriptional remodeling. We postulate that the disturbance of the potential mitochondrial to nuclear signaling pathway causes iron accumulation in mitochondria and relative iron deficiency in cytosol [39]. Similar to the effects caused by many mitochondrial Fe-S synthesis defects, GLRX5 deficiency also results in mitochondrial iron overload [74, 81]. The IRE- binding activities of IRP proteins are elevated, which in turn inhibits the translation of mRNAs that contain 5′IREs. In addition, GLRX5 is intimately involved in erythropoiesis as revealed by the following results [81]; first of all, GLRX5 is highly expressed in bone marrow and particularly in erythroid precursor cells, as shown by both bioinformatics data and *in situ* hybridization; secondly, GLRX5 expression is induced during erythroid differentiation, in association with two other heme synthesis proteins, ALAS2 and ferrochelatase [82, 83]; and thirdly, GLRX5 deficiency caused a sideroblastic anemia in a human patient [74] and anemia in zebrafish [76]. Finally, GLRX5 deficiency decreases heme synthesis and hemoglobinization [81]. During erythroblast differentiation, ALAS2, ferrochelatase and GLRX5 expression are all upregulated in order to synthesize large amounts of heme [81]. We postulate that GLRX5 and other essential mitochondrial Fe-S biosynthesis proteins continue to produce and export a factor that signifies the mitochondrial iron homeostasis is normal. General cellular iron homeostasis is not disturbed, and IRE binding activity of IRP1 remains low. ALAS2 mRNA that contains a 5′ IRE is therefore translated into protein adequately. Based on the facts that ferrochelatase protein is readily degraded in the absence of its Fe-S cluster [82] and that ferrochelatase protein levels are substantially decreased in GLRX5 deficient patient lymphoblasts [81], we have hypothesized that GLRX5 may deliver the preassembled [2Fe-2S] cluster to ferrochelatase. Thus, normal GLRX5 activity allows expression of ALAS2 and ferrochelatase to increase during hemoglobinization for erythropoiesis [81]. On the other hand, GLRX5 deficiency leads to a transcriptional increase of ferroportin (FPN1) mRNA, including the FPN1a and 1b forms [81]. The FPN1b mRNA is expressed in erythroblasts and duodenal mucosal cells. It does not contain a 5′IRE, and IRP proteins therefore cannot repress its translation [84]. The increased FPN1 protein levels in erythroblasts probably lead to severe cytosolic iron deficiency. This erythroid-specific expression of two novel transcripts, the IRE-containing ALAS2 and the nonIRE containing FPN1b, may explain why the phenotype of GLRX5 deficiency manifested itself mainly in the erythroid tissues of a human patient [81]. 

 Isa1 and Isa2, the yeast homologues of ISCA1 and ISCA2, are thought to function as alternative scaffold proteins that can substitute for the Isu scaffold in the mitochondrial Fe-S cluster biogenesis of yeast [51]. It was recently discovered that expression of these putative scaffold proteins is induced in animals when heme biosynthesis enzymes are induced [83]. In yeast, Isa1 and Isa2 may receive an Fe-S cluster intermediate from Isu, the dominant scaffold, and assemble Fe-S clusters for a subgroup of target proteins [85], including mitochondrial aconitases and several enzymes of the radical S-adenosylmethionine (SAM) Fe-S protein family, including biotin synthase and lipoate synthase in yeast [85]. The radical SAM superfamily comprises more than 2800 proteins that chelate [4Fe-4S] clusters. These enzymes use the strong reducing potential of a low potential [4Fe-4S] cluster to generate a powerful oxidizing agent, the 5′-deoxyadenosyl radical, from SAM, for review see [86]. A newly identified yeast protein, Iba57, facilitates this process by physically interacting with Isa1-2. Although it is still unknown how they work, Isa1, Isa2, and Iba57 form a complex and assemble Fe-S clusters for mitochondrial aconitase and radical SAM Fe-S proteins in yeast [63]. 

 In addition, a recent large-scale gene expression analysis has revealed that ISCA1 and ISCA2, the human homologues of Isa1 and Isa2, and the human Iba57-like protein, C1orf69, are required for heme biosynthesis in animals [83]. Experimental results have demonstrated that, similar to other heme synthesis enzymes, ISCA1 and C1orf69 are induced during erythroid differentiation. *In situ* hybridization analysis using zebrafish embryos shows that C1orf69 is specifically expressed in hematopoietic tissues. Knockdown of either ISCA1 or C1orf69 reduces heme production and hemoglobinization, and thus causes profound anemia in zebrafish. Although the mechanism is not known, it is hypothesized that ISCA1-2 and C1orf69 may assemble Fe-S clusters for mitochondrial aconitase and for lipoate synthase, which may produce a cofactor for the pyruvate dehydrogenase complex (PDC) [83]. Both PDC, the enzyme responsible for converting pyruvate to acetyl-CoA to enter the citric acid cycle, and aconitase, a citric acid cycle enzyme, are directly involved in producing succinyl-CoA, a substrate in the first step of heme synthesis catalyzed by ALA synthase, in which succinyl-CoA and glycine are condensed into the product, 5-aminolevulinate. Their potential effects on succinyl-CoA synthesis might partially explain why and how ISCA1-2 and C1orf69 proteins are important in heme biosynthesis (Figure 1). Even though Isa and Iba57 have been shown to be required for activity of lipoate synthase in yeast [63], it is not known whether ISCA and C1orf69 have similar roles in activating PDC of heme-synthesizing animals. 

 Along with their findings about ISCA and C1orf69, investigators also identified other candidate genes that are essential for heme synthesis [83], including SLC25A39, which is orthologous to yeast MTM1. This protein is implicated in mitochondrial iron homeostasis, and its mutation leads to misincorporation of iron to Mn-SOD and iron overload in mitochondria [87–89]. Based on the phenotypes of MTM1 and GLRX5, the authors predicted that SLC25A39 may be involved in Fe-S biogenesis. Furthermore, this large- scale expression study has generated a collection of candidate genes that are probably required for heme biosynthesis [83], some of which may be uncharacterized Fe-S biogenesis genes in erythroblasts. 

 In summary, Fe-S biogenesis is required to execute several steps of heme synthesis, particularly when large amounts of heme are needed for hemoglobinization during erythropoiesis. Multiple heme synthesis enzymes definitely need Fe-S for activity, such as ferrochelatase whereas some others need Fe-S cofactors to potentially provide substrates for heme synthesis, for example PDC and mitochondrial aconitase [83], and others such as ALAS2 can be efficiently translated only when cytosolic Fe-S biogenesis is intact. In principle, since the Fe-S biogenesis pathway involves at least 20 proteins, any of these Fe-S biogenesis proteins may impact heme synthesis. But so far only GLRX5, ABCB7, and the potential ISCA1-C1orf69 complex are clearly demonstrated by hematopoietic phenotypes in model systems and diseases to be important in heme synthesis. Interestingly, they (GLRX5, ABCB7, ISCA1 and C1orf69) are all highly expressed in hematopoietic tissues, compared to other Fe-S synthesis proteins which are more evenly distributed among many tissues, such as ISCS and ISCU. Moreover, all of them except ABCB7 are induced during erythroid differentiation, similar to the heme synthesis enzymes.
